# Enzymatic and acid conversion of new starches from improved orphan crops: prospects for renewable materials uses in food and non-food industries

**DOI:** 10.1186/2193-1801-3-498

**Published:** 2014-09-03

**Authors:** Ginette Doué, Micaël Bédikou, Gisèle Koua, Rose-Monde Mégnanou, Sébastien Niamké

**Affiliations:** Laboratoire de Biotechnologies, Filière Biochimie-Microbiologie, Unité de Formation et de Recherche en Biosciences, Université Félix Houphouët-Boigny, 22 BP 582 Abidjan 22, Abidjan, Cocody, Côte d’Ivoire

**Keywords:** Orphan crop, Modified starch, Biodegradable material, *Manihot esculenta*

## Abstract

The enzymatic and acid hydrolysis have converted eight new starches into a range of chain lengths mainly including glucose, maltose, and maltodextrins as observed on TLC plates, irrespective to the starch variety and treatment. Results of the enzymatic hydrolysis have highlighted the possibility of the use of V_4_ and V_64_, which can be labelled as “dietary fibres”, to enhance the organoleptic qualities of foods and for fibre fortification of low-calorie products. Concerning V_66_ and V_69_, they have much relevant in food, textile and pharmaceutical applications. The acid hydrolysis showed that V_73_ is the best starch in the chemical industry for making environment-friendly products such as plastics. Because starch is a natural component that degrade quickly in normal composting condition, the whole studied starches could be advised for various utilizations in the food, textile, paper, biofuel, pharmaceutical and plastic industries for sustainable development.

## Introduction

Orphan or neglected crops are categorized under main crop types such as, cereals, legumes, fruit and root crops. They are considered as staple food crops in many developing countries by contributing to the diet of a large portion of resource-poor consumers (Ravi et al. 2010). So far, the world food supply has been dependent on few of these crop plants mainly cereals. But today, the cereal deficit to meet global food requirements has encouraged the diversification of crops for both food and non-food productions. So, in some tropical climate regions, root and tuber crops have got attention to contribute a part in feeding the world. In Africa for example, cassava was commonly referred to, as the cornerstone of food security.

Initially grown as a substantial food crop, cassava (*Manihot esculenta* Crantz) is one of the most important food crops in the humid tropics, being particularly suited to low nutrient availability and able to survive drought (Burrell 2003). Because it is such an important crop, the competing needs for cassava cut across both human and animal consumption (Oppong-Apane 2013). Outside these aspects, the growing interest in root crops is partly due to their high starch content that is an appropriate, inexpensive and commodity raw material for sustainability.

Starch is next to cellulose and chitin, the most important carbohydrate in the plant system (Tharanthan 2005). Structurally, it is mainly composed of two polysaccharides namely amylose and amylopectin which are enclosed in microscopic particles called granules (BeMiller 1997). As not only the main source of food for human, starch is a renewable polymer widely used in an impressive range of products in food and non-food industries. As a biodegradable material with controllable lifetimes, the attention paid to starch is growing as the years pass. Indeed, starch is increasingly used as substitute for non-renewable fossil fuel in plastics production (Singh et al. 2010; Akpa and Dagde 2012). Elsewhere, in medicine for example, four new types of starches called resistant starches (RS), are used to solve problems of nutrition in diabetes and other related diseases (Nugent 2005). Resistant starches are defined as starches or starch degradation products that escape from digestion in the small intestine of healthy individuals. These starches can be obtained in their nature state (RS type I or II) in whole seeds, legumes, grains, unripe bananas and potatoes (Moongngarm 2013), or by retrogradation of amylose molecules (RS III) after moisten, heating and cooling treatments (Eerlingen et al. 1993). The last type of resistant starch are obtained by chemical modification (RS IV) and could have a wide variety of structures not found in nature (Fuentes-Zaragoza et al. 2010). In view of all these potentialities, it seems obvious that nowadays, practically every industry in existence uses starch or its derivatives in one form or another for specific applications.

Although starch has many sources, the most important starches produced and used in developing countries were obtained from corn, wheat and potato. However, in countries with high starch utilization like the United States, cassava has also been the traditional sources of starch used mainly in the food industry due to its particular and interesting physicochemical and functional properties (Takizawa et al. 2004; Che et al. 2007). Such particular properties have made cassava starch a commodity renewable material in various industrial sectors, and is now a preferred material for making biofuels (Jansson et al. 2009). Cassava native starch can be physically, chemically or enzymatically modified or derivatized to reach technical and specific needs. For example, it is a raw material for glucose, maltose and maltodextrins production which in turn are used as textural agents by the confectionery industry. In Vietnam, cassava utilization in the food industry had largely been reported (Goletti et al. 2001). Finally, cassava starch has many beneficial characteristics that lead its use as an input to a wide range of products in various industrial sectors such as foods, paper, textile, pharmaceutics and construction (Che et al. 2007; Demiate and Kotovicz 2011). As the African continent is concerned, this trend has only just started to take initial shape.

Even though starches isolated from different sources have widely functional properties, there is a continuously rising demand for new and improved specific functional traits. As already reported by Burrell (2003), any significant or minor improvement of a starchy crop could result in important societal impact as a result of the huge volumes of starch consumed globally. Recently, the national agricultural centre of Côte d’Ivoire (CNRA) has implemented for rural extension, new improved cassava varieties with particular traits (high yield and yellow coloured fleshes). Prior to their extension, flours and starches of some of them were extensively characterized by our Laboratory through physicochemical, biochemical and functional properties (Mégnanou et al. 2013; Doué et al. 2013, 2014). In the present paper, eight (8) of these types of starch were extracted and subjected to amylolytic enzymes and hydrochloric acid conversion. Their behaviour toward both hydrolysis are discussed in term of potential industrial applications.

## Results and discussion

Unprocessed native starches are structurally and functionally too restricted for application in todays advanced technologies. So, processing is necessary to engender a range of functionality. It could be biochemical or chemical.

### Biochemical modification (enzymatic hydrolysis)

**Figure 1:**
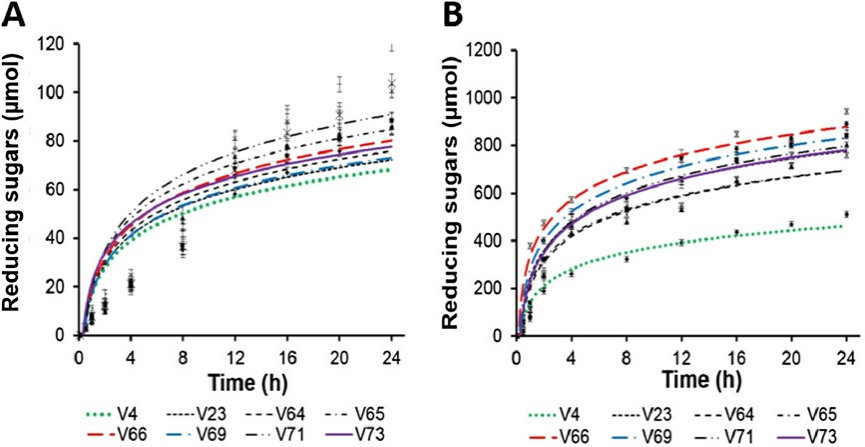
**Kinetics of enzymatic hydrolysis at 37°C of native (A) and gelatinized (B) cassava starches by the digestive fluid of the giant snail** ***Achatina achatina*** **.**

The kinetics of the enzymatic hydrolysis at 37°C of both native and gelatinized starches are shown in Figure 1. These profiles obeyed the Michaelis-Menten kinetics which are characterized by logarithmic curves. So, the earlier linear rising sides of these profiles allowed us to determine the initial velocities topped in Table 1.

**Table 1:** **Initial velocities of enzymatic hydrolysis of native and gelatinized starches by the digestive fluid of the giant snail** ***Achatina achatina***

Starch varieties	Initial velocity (UI/mg of proteins)	
	Native starch (10 ^−2^)	Gelatinized starch (10 ^2^)
**V**_**4**_	8.12 ± 0.08	1.57 ± 1.01
**V**_**23**_	10.37 ± 0.12	1.14 ± 0.84
**V**_**64**_	7.90 ± 0.06	2.12 ± 1.89
**V**_**65**_	11.48 ± 0.07	2.21 0.93
**V**_**66**_	11.37 ± 0.11	6.32 ± 1.53
**V**_**69**_	9.14 ± 0.09	3.47 ± 0.78
**V**_**71**_	8.88 ± 0.08	2.06 ± 1.33
**V**_**73**_	18.77 ± 1.03	1.82 ± 0.79

Values are slopes of the earlier linear portion of the kinetic curves of product formation as a function of time depicted in Figure 1.

The initial velocities are the expression of each starch sturdiness and traduce its ability to be greatly hydrolyzed or not. The results indicated that, up to 24 hours of incubation time, and irrespective of variety kind, native starches were less susceptible to enzymatic hydrolysis than the gelatinized ones. This difference of behavior could result in the modification of the internal structure of native starch granules during hydrothermal treatments as already verified by Amani et al. (2004) and Chung et al. (2006).

For native starches isolated from various varieties of cassava, the lowest values (Table 1) of initial velocities of enzymatic hydrolysis were observed with V_64_ and V_4_ (about 7.90 and 8.11 × 10^−2^ UI/mg of proteins), respectively. While V_73_ showed the highest (18.77 ± 1.03 × 10^−2^ UI/mg of proteins) initial velocity. Concerning gelatinized starches, it is V_66_ which was greatly hydrolyzed (6.32 ± 1.53 × 10^2^ UI per mg of proteins) followed by V_69_ (3.47 ± 0.78 × 10^2^ UI / mg of proteins).

The great tendency to hydrolyse gelatinized starches V_66_ and V_69_ was probably due to their higher water solubility in agreement with the data of Doué et al. (2013). The improvement of starch paste clarity is much appreciated by food and textile industries. So, the great hydrolysed starches, reported in this study such as V_66_ and V_69_ could serve as suitable materials in the above cited industries. For example, they can be used in pastry as fillings for cakes. Thus, it is obvious that these starch varieties have predispositions for other industries and in particular, desirable qualities which could make them widely used in food products preparation.

Contrary to V_66_ and V_69_, the already disseminated cassava gelatinized starch (V_23_ and V_4_) have both showed lower amylolytic enzymes attack with initial hydrolysis velocities of about 1.14 and 1.57 × 10^2^ UI per mg of proteins, respectively. In addition, V_4_ was less hydrolyzed either in its native or gelatinized form. The high robustness of the native starches (V_64_ and V_4_) and the gelatinized starches (V_4_) face to enzymes attack reminds us of a new type of starches called resistant starch type II (RS II). These kind of starches are, in an uncooked state, naturally resistant to enzymes attack. As far as gelatinized starches are concerned, V_4_ could be classified as RS III or IV which are formed through retrogradation of higher amylose starch variety such they are no longer well recognised by alpha-amylases thus less hydrolysed. The relatively high amylose content of V_4_ (25.31%) compared with this of the other starches (amylose content ranged from about 16 to 20%) as verified by Doué et al. (2014), enable us to corroborate the above assertion. It was previously reported that resistant starches are healthy diets. By providing low water-holding capacity thereby aiding processing, RS which could also be labelled as “dietary fibres” are used to enhance the organoleptic qualities of food as ingredients for the fibre fortification of low-calorie products (Croghan 1998). Also, resistant starch consumption offers a controlled rate of blood glucose as compare to highly-processed carbohydrates. Although research on the benefits of resistant starches are recent, it has been already reported that they have potential physiological benefits for diabetics (Nugent 2005).

### Chemical processing (Acid hydrolysis)

**Figure 2:**
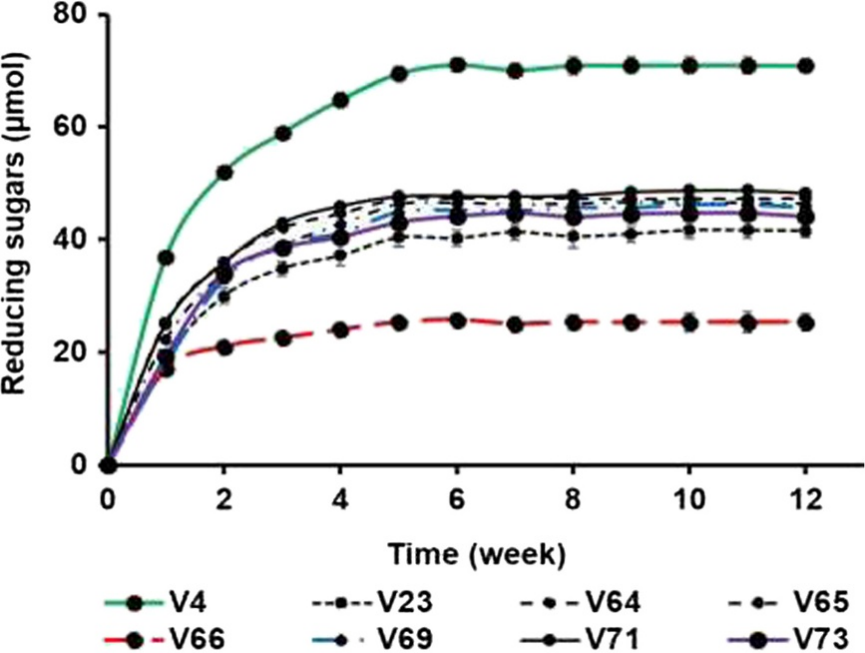
**Kinetic of 2.2 mol.l** ^**−1**^ **hydrochloric acid hydrolysis at 25°C of native cassava starches varieties.** Reactions were carried out for 3 months.

The studied starch varieties have been subjected to mild hydrochloric acid (2.2 mol.l^−1^) hydrolysis in order to explore the possible areas of their utilisation. Results of this study are depicted in Figure 2 which shows the predominant hydrolysis of the native starch V_4_. In contrast, V_66_ was weakly attacked by the acid solution. The other native starches showed an intermediate tendency to hydrochloric acid hydrolysis. The two-stage of hydrolysis pattern was quite obvious in all cases. A relatively fast hydrolysis rates during the first two weeks which is linked to the depolymerisation of the amorphous regions of starch granules while the crystalline material is slowly degraded during the second stage (Robin et al. 1974). The best thin-boiling or fluidity starches are characterized by an increase in ratio of smaller and linear molecules which develop strong gels on cooling (Taggart 2004). The strong gelling property of acid-thinned starches lead to their use as thickener and ambient products stabilizer. Such starch properties, with close resemblance to the best native fluidity starch V_4_ (Figure 2), are preferentially sought in the preparation of glues and gums, and in the paper and textile industry for coating and sizing. Lower viscosity acid-thinned starches such as V_23_, V_64_, V_65_, V_69_, V_71_ and V_73_ which were, next to V_4_, greatly converted to starch derivatives could also be used in the textile industry to protect yarn during weaving and in the paper industry to improve printable paper qualities (Lim et al. 1992).

### Thin layer chromatography

**Figure 3:**
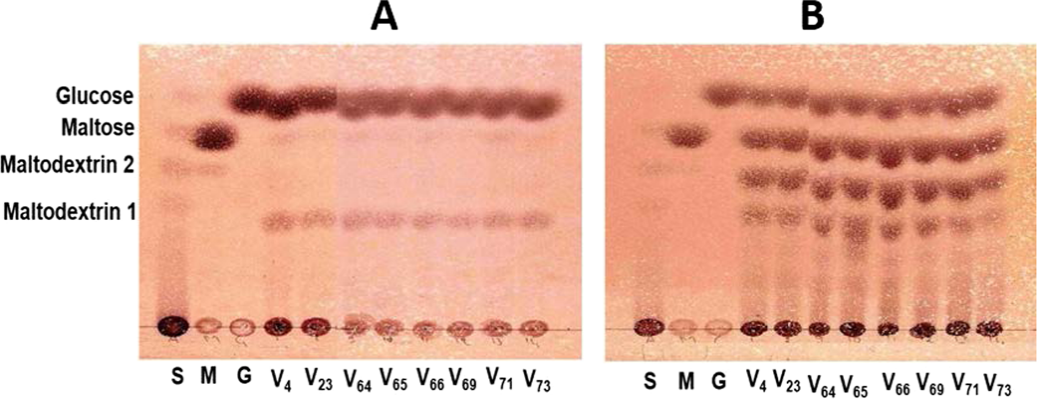
**Thin layer chromatography of products from 5 μl reaction mixture of native (A) and gelatinized (B) cassava starches catalyzed by the digestive fluid of the giant snail** ***Achatina achatina*** **.** S, M and G are standards of starch, Maltose and Glucose. V_4_, V_23_, V_64_, V_65_, V_66_, V_69_, V_71_ and V_73_ are isolated starches from cassava varieties.

On thin layer chromatography plates, glucose and maltodextrin 1 were distinctly observed as the common products from both native and gelatinized starch substrates (Figure 3). In addition to the latter products, maltose and another maltodextrin with relatively lower molecular mass (Maltodextrin 2) were obtained for gelatinized starches (Figure 3B). Glucose, maltose and maltodextrins are essential and useful products for the food confectionary industries (Zainab et al. 2011; Akpa and Dagde 2012). They are widely applied in sugar, spirits, and textile as well as in brewing industries. For example, glucose syrup could serve as substrates for the production of a wide range of fermentation products such as monosodium glutamate (Nampoothiri and Pandey 1999), lysine for the animal feed industry (Moosavi-Nasab et al. 2007) and ethanol for biofuel industry (Duhan et al. 2013). Dextrins are used in backed goods and in confectionary where their main functions are viscosity control, softening, texturing and coating (Gunaratne et al. 2007; Akpa and Dagde 2012).

The concept of sustainable development trotted the past few years is related to the use of renewable energies sources as starch. In this context, the whole studied cassava starches, and particularly those which have shown great susceptibility to enzymes conversion such as V_73_ (on its native form) and V_66_ (on its gelatinized form), can be used as feedstock. Such biodegradable materials could also serve for the production of a vast array of safe carbon-based products including biofuels and plastics on which the modern society mainly depends (Šárka et al. 2011). A particular attention should be pay to native (V_73_) and gelatinized (V_66_ and V_69_) starches for their potential use as binder in the pharmaceutical industry. Indeed, cassava starch has previously been reported to have very good tablet excipient properties mainly based on its adhesive, thickening, gelling, swelling and forming characteristics (Kunle et al. 2003; Kulkarni et al. 2010).

## Conclusion

It could be conclude that the whole studied cassava starches have shown different hydrolysis pattern toward amolytic enzymes of the digestive fluid of the giant snail *Achatina achatina,* and hydrochloric acid. In their native form, the best enzymatic hydrolysis was observed with V_73_ as substrate (18.77 ± 1.03 × 10^−2^ UI/mg of proteins) while V_64_ and V_4_ recorded the lowest values of initial velocity of around 8 × 10^−2^ UI per mg of proteins. Therefore, before thermal treatment, native starches V_4_ and V_64_ could be recommended as RS. In their gelatinized form, only V_4_ showed RS characteristics face to amylolytic enzymes. In contrast, native starch V_4_ was predominantly hydrolysed in the presence of the hydrochloric acid solution while V_66_ was weakly attacked. After a thermal treatment, gelatinized starches were better hydrolysed but not necessarily in the same trend. Analysis of hydrolysis products on TLC plates have revealed the presence of glucose, maltose, and maltodextrins which are essential and useful products in the food industry. Moreover, the basic knowledge gained from this study would be useful information and led to the possibility of suggesting some of these new types of starch in an impressive range of industry such as foods, textiles, papers, biofuels and pharmaceutics. However, much investigation is still required to fully understand the application of these raw materials.

## Materials and methods

### Cassava varieties

All the improved varieties of cassava used in this study were harvested a eleven years old and were provided to us by the National Agricultural Research Centre (CNRA, Adiopodoumé, Côte d’Ivoire 5°19′40″ latitude North and 4°23′00″ longitude West). They were encoded V_64_, V_65_, V_66_, V_69_, V_71_ and V_73_, and resulted from various crossbreeding’s of two local and already disseminated varieties: V_4_ (white coloured flesh) and V_23_ (yellow coloured flesh). In this report, the identification codes of cassava varieties will also serve for their different starches.

### Enzymatic source and amylolytic enzymes extraction

Some giant snails *Achatina achatina* were kept for three days without eating or drinking in order to liquefy their initial bolus. Then, their shells were cracked and the digestive fluid was collected with syringe. The crude extract was filtered through sterile cotton wool and then centrifuged at 10,000 g for 15 min. The supernatant was used as the source of amylolytic enzymes.

### Proteins estimation

Proteins concentration in the crude extract was measured by using the Folin-Ciocalteu’s phenolic reagent following the experimental protocol described by Lowry et al. (1951). Bovine serum albumin was used as the standard protein.

### Starch extraction

Fresh cassava roots were washed, peeled, chopped into approximately 1 cm cubes and disintegrated by a high speed blender for 5 min. Then, the cassava mash was suspended in ten times its volume of water, stirred for 5 min and filtered using double fold cheesecloth. The filtrate was allowed to stand for 2 h and the top liquid was decanted and discarded. A 4% (w/v) NaCl solution was added to the sediment and the mixture stirred for 5 min. Filtration was carried out using sieves with reverse mesh sizes varying from 500 to 100 μm. After decanting the top liquid, starch paste was dried at 45°C for 48 h, crushed to a fine powder and then stored for further treatments.

### Native and gelatinized starch substrate preparation

Native starch substrates were obtained by dissolving 1 g of the above dried powder in 100 ml of distilled water. For gelatinized starches, 1 g of dried powder was initially suspended in 50 ml of distilled water. Then, the mixture was heated at 70°C for 10 min and the residual solution adjusted to 100 ml with distilled water.

### Enzymatic and mild-acid hydrolysis

The enzymatic hydrolysis were performed on both starch substrates. Reactions were carried out at 37°C with 2.5 μg of proteins in a 100 mM sodium acetate buffer (pH 5.6) for 24 hours. Concerning the mild acid hydrolysis, only native starches were used. A 2.2 N hydrochloric acid solution was used at 25°C to catalyse reactions for three (3) months. All the experiments were performed in triplicate under sporadic stirring with a total mixture volume of 200 μl by using a screw-cap glass cells. Amylolytic enzymes activities were expressed as the amount of catalysts which release 1 μmol of reducing sugar from 1% (w/v) starch in a minute under the experimental conditions. For the acid conversion, activities were expressed in percent (mg of dextrose equivalent per 100 mg of starch). The initial velocities were graphically determined from the beginning rising side (linear part) of each hydrolysis profile by taking into account the earlier upward trend of reducing sugar production.

### Analysis of hydrolysed products

At regular time intervals, 100 μl volume samples were withdrawn to quantify total reducing sugars (Bernfeld 1955), while 5 μl of the same solution were spotted onto thin layer chromatograph plates (Model 60 F254, Merck) to monitor the hydrolysis of native and gelatinized starches. TLC plates were run with butanol-acetic acid-water mixed at 9:3.75:2.25 (v/v/v), and sprayed with a naphto-resorcinol in ethanol and a H_2_SO_4_ 20% (v/v) solution. Then, sugar spots were visualized by heating plates at 110°C for 5 min.
